# A feasibility study of a best practice health literacy app for Australian adults with chronic kidney disease

**DOI:** 10.1016/j.pecinn.2022.100047

**Published:** 2022-05-05

**Authors:** Stephanie Zwi, Jennifer Isautier, Angela C. Webster, Kelly Lambert, Heather L. Shepherd, Kirsten J. McCaffery, Kamal Sud, John Saunders, Emma O'Lone, Na Liu, Jinman Kim, Aphra Robbins, Danielle Marie Muscat

**Affiliations:** a Sydney Health Literacy Lab, Sydney School of Public Health, Faculty of Medicine and Health, The University of Sydney, NSW, Australia; b Sydney School of Public Health, Faculty of Medicine and Health, The University of Sydney, NSW, Australia; c NHMRC Clinical Trials Centre, Faculty of Medicine and Health, University of Sydney, NSW, Australia; d Westmead Applied Research Centre, Westmead Hospital, Westmead, NSW, Australia; e School of Medicine, Faculty of Science, Medicine and Health, University of Wollongong, NSW, Australia; f Faculty of Medicine and Health, The University of Sydney, NSW, Australia; g Nepean Clinical School, Faculty of Medicine and Health, The University of Sydney, NSW, Australia; h Department of Renal Medicine, Nepean Hospital, Nepean Blue Mountains Local Health District, NSW, Australia; i Department of Renal Medicine, Royal Prince Alfred Hospital, Sydney, Local Health District, NSW, Australia; j Department of Renal Medicine, Royal North Shore Hospital, Northern Sydney Local Health District, NSW, Australia; k School of Computer Science, Faculty of Engineering, The University of Sydney, NSW, Australia; l Telehealth and Technology Centre, Nepean Hospital, Nepean Blue Mountains Local Health District, NSW, Australia; m Consumer representative, Sydney, NSW, Australia

**Keywords:** Chronic kidney disease, Health literacy, Shared decision making, E-health, Smartphone app, Ethnic groups

## Abstract

**Objective:**

To investigate feasibility of the SUCCESS app; a cross-platform e-health innovation to improve health literacy, self-management and shared decision-making among culturally-diverse Australian haemodialysis patients.

**Methods:**

Multi-site, pre-post, mixed-methods study. Haemodialysis patients ≥18 years used the app for 12 weeks. Qualitative data from 18 interviews were thematically analysed to evaluate app acceptability. Quantitative analysis using paired sampled *t-tests* evaluated feasibility outcomes pertaining to recruitment, retention, data collection and app efficacy (including health literacy; decision self-efficacy; quality of life; behaviour; knowledge; confidence).

**Results:**

We successfully recruited diverse participants (*N* = 116; 45% born overseas; 40% low/moderate health literacy) from four Local Health Districts in Sydney, Australia. However, only 61 participants completed follow-up questionnaires. Qualitative analyses provided insights into acceptability and user engagement. Quantitative analyses showed significant improvements on the health literacy domain *‘Ability to actively engage with healthcare providers’* (Mean Difference [MD] = 0.2 on a 5-point scale; CI_95%_: 0.0–0.4; *p* = 0.03) and decision self-efficacy (MD = 4.3 on a 10-point scale; CI_95%_: 0.6–7.9; *p* = 0.02) after 12 weeks app use.

**Conclusions:**

The SUCCESS app was feasible and acceptable to participants. The app will be adapted to facilitate ongoing use and engagement among diverse haemodialysis patients.

**Innovation:**

This is the first health literacy-informed app to promote active participation in haemodialysis self-management and decision-making, tailored toward culturally-diverse and low health literacy groups.

## Highlights

• The SUCCESS app was feasible and acceptable to culturally-diverse dialysis patients
• Health literacy and decision self-efficacy scores improved significantly
• No changes were seen in quality of life, behaviour, knowledge or confidence scores
• Practical health information was accessed more than skills training within the app
• Participants provided useful suggestions to promote ongoing engagement with the app

## 1 Introduction

In 2017, chronic kidney disease (CKD) resulted in 1.2 million deaths and was the 12th leading cause of mortality worldwide [1]. In developed countries such as Australia, culturally and linguistically diverse populations bear a disproportionate burden of CKD with greater prevalence, higher mortality and accelerated disease progression [2]. Limited health literacy is common in CKD; a systematic review conducted in 2017 found the pooled prevalence of limited health literacy among people living with CKD to be 25%, with significant between-study heterogeneity and higher prevalence among individuals with low socioeconomic status and non-white ethnicity [3]. Available evidence suggests associations between lower health literacy and adverse clinical events, increased healthcare use and mortality in the context of CKD [4]. While educational programs for people with CKD requiring dialysis are now standard practice across Australian hospitals and dialysis centres, a recent Cochrane review demonstrated that there are currently no models of care which promote global health literacy skills applicable across multiple decision contexts, tailored to ethnically-diverse and lower literacy patient groups [5].

Optimal management of CKD is complex and multifaceted, and includes fluid and dietary restrictions, particularly for salt, potassium and phosphate. Haemodialysis patients experience a high symptom burden of fatigue, itch and muscle cramps alongside deficits in memory, cognition and executive function contributing to reduced quality of life [6]. Self-management and shared decision-making are important predictors of CKD outcomes [7], yet low health literacy remains a barrier to actively engaging in these behaviours. A scoping review of reviews conducted in 2021, for example, found that patients with limited health literacy experience difficulties with specific domains of self-management, particularly in the areas of medical management, communication and knowledge [8]. Information technology tools including mobile apps, web-based portals, and web-based educational or coaching interventions are increasingly being adopted to support disease self-management, and growing evidence links their usage to improved clinical outcomes [[9], [10], [11], [12], [13], [14], [15]]. However, very few available apps support self-management specifically for people with CKD requiring dialysis [16], and none have been informed by health literacy theory.

Our team developed a cross-platform smartphone application (the “SUCCESS app”) to support Australian adults with CKD requiring dialysis to actively participate in self-management, improve health literacy and engage in decision-making. The app used a two-pronged health literacy approach by adopting strategies to reduce the complexity of content related to diet, fluids, medicine, physical activity and supportive care; and including features to improve communicative and critical health literacy skills. The latter included question prompt-lists and evidence-based volitional help sheets to support question-asking and behaviour change, as well as animated skills training related to communication, shared decision-making and critical appraisal of health information. Full details of the content and app development has been published elsewhere [17].

Here, we detail the results of a feasibility study of the SUCCESS app conducted in New South Wales (NSW), Australia during 2019–20. The importance of feasibility studies for optimising complex health interventions and evaluation designs before evaluating effectiveness is widely acknowledged [18,19]. In this instance, the need for a feasibility study was indicated by a number of factors [20] including the unique nature of the intervention (this is the first health literacy-informed app developed to promote active patient participation in CKD management and decision-making), and the diverse and understudied population group.

We intended results of this feasibility study to inform a definitive trial of this intervention, as well as trials of similar interventions in the future. Our specific objectives were to evaluate: (i) recruitment capability and resulting sample characteristics; (ii) data collection procedures and outcome measures; (iii) acceptability and suitability of the SUCCESS app and study procedures; (iv) efficacy of the SUCCESS app intervention.

## 2 Methods

### 2.1 Study design

We conducted a multi-centre feasibility study using a pre-post design and mixed-methods evaluation. Approval for this study was obtained from the Nepean Blue Mountains Local Health District Human Research Ethics Committee (HREC/18/Nepean/109).

### 2.2 App development

The SUCCESS app was co-developed with a multidisciplinary team including public health researchers, renal clinicians, allied health professionals and consumers living with kidney disease [17]. The app included two core components; a ‘Health Information’ section to support functional health literacy and a ‘Skills for Health’ section to develop communicative and critical health literacy skills (see Box 1 for more details).

Box 1 **Content areas and features of the SUCCESS app.**

**Table:** 

**Health Information**	**Skills for health**
Diet a) Important nutrients b) Eating out c) Managing symptoms d) Reading food labels e) How to modify recipes	Talking to your healthcare team a) Providing information b) Asking questions c) Checking you understand d) Expressing how you feel
Medicine a) What do I need to check before I take my medicine? b) Active ingredients c) When to use medicine d) Medicine dosage and timing e) Expiry dates f) Reviewing my medicines g) Pharmaceutical Benefits Scheme	Making decisions a) Say what you prefer b) Ask about your options c) Tell the team about yourself d) Think about what is best for you
Supportive care a) What is supportive care? b) What happens after stopping dialysis? c) What is advance care planning?	Can I trust this health information? a) Questions to ask when reviewing health information i. Who said it? ii. When did they say it? iii. How did they know?
Fluids a) What counts as a fluid? b) How much fluid is in my food? c) What happens when I have too much fluid? d) Keeping track of my fluid e) Tips for taking my medications	
Physical activity a) What is physical activity? b) Addressing concerns about physical activity c) What physical activity can I do? d) When to stop exercising

Alt-text: Box 1

We used a four-step process to simplify written content, including calculating readability statistics, reducing readability to a maximum of US grade eight level [21], supplementing written information with audio-visual content, and incorporating micro-learning and interactive quizzes [17]. Question prompt lists (to support question-asking and patient engagement in healthcare consultations) and volitional help sheets (behaviour change tools involving “if-then” plans) were designed for each content area to increase question-asking and guide behaviour change, respectively [17].

The ‘Skills for Health’ section included a series of short animations related to health literacy skills including doctor-patient communication, shared decision-making and critical appraisal of online health information. These were designed specifically for the SUCCESS app, based on evidence to suggest the utility of spoken animation for adults with low health literacy, and underwent a similar process of simplification to the written content [22].

### 2.3 Recruitment of participants

We invited haemodialysis patients aged 18 years and over to participate. We recruited from in-patient and out-patient dialysis centres at five hospitals across four metropolitan Local Health Districts (LHDs) in NSW, Australia.

To ensure representation from a broad range of cultural, linguistic and educational backgrounds, LHDs were purposively sampled to reflect the geographic and socio-demographic diversity of dialysis patients in NSW. Our intent beyond this study is to translate and culturally adapt the app into multiple languages. However, for this feasibility groundwork, participants were required to read and speak sufficient English to respond to questionnaires.

Eligible participants were identified by dialysis nurses and approached by researchers during their scheduled dialysis sessions. Participants who did not own a smartphone or tablet were invited to use tablets provided by the research team made available during dialysis sessions. Recruitment logs and field notes were used to record the number of participants recruited at each site, time taken to achieve recruitment targets and challenges associated with recruiting participants during scheduled dialysis sessions. These were analysed thematically to identify broad challenges, and to troubleshoot the recruitment process appropriately.

Once informed consent was obtained, participants completed baseline questionnaires with optional assistance from a researcher or caregiver. Questionnaires captured demographics (age, gender, country of birth, years spent living in Australia, Aboriginal and Torres Strait Islander status, education), clinical characteristics (years on dialysis), and baseline levels of health literacy, cognitive impairment and digital literacy (Table 1).

**Table 1:** Assessment of baseline health literacy, cognitive impairment and digital literacy in the SUCCESS feasibility study.

Measure	Description
Health literacy	*Self-report:* The Brief Health Literacy Screener (BHLS) is a single-item screener to identify people with inadequate levels of functional health literacy in clinical settings [23,24]. The question asks, *‘How confident are you filling out medical forms by yourself?’,* with a five-point likert scale ranging from 1 = *not at all* to 5 = *extremely*. The threshold for inadequate health literacy is 3 = *somewhat* or less. *Performance-based:* A brief four-item comprehension test based on instructions similar to those found on a packet of Aspirin bought over the counter [25]. Participants are asked to read a fictitious medicine label and respond to four questions, such as *‘What is the maximum number of days you may take this medicine?’* and *‘List one condition for which you might take the tablet’.* Patients score 1 point for each correct answer. Health literacy is categorized as high (4/4 correct answers), medium (3/4), or low (≤2/4) [25].
Cognitive impairment	The Montreal Cognitive Assessment (MoCA) is a 10-min cognitive screening tool [26]. It measures a person's orientation to time and space, short-term memory, abstract reasoning, attention and other aspects of cognitive ability. MoCA returns a cognitive ability score ranging from 0 to 30 where a score of 26 or below suggests cognitive impairment.
Digital literacy	The assessment of digital literacy was adapted from a validated instrument, the Multicomponent Assessment of Computer Literacy (MACL). The MACL is a seven-point likert scale consisting of statements about attitudes towards computers [27]. For the purposes of this study, the questions were modified to reflect the context of smartphone usage such as *‘I am able to download apps to my phone independently’.*

### 2.4 Data collection procedures and outcome measures

Outcomes were assessed at baseline and 12 weeks follow-up, using questionnaires completed during dialysis sessions. Outcome measures included change in health literacy skills, decision self-efficacy, quality of life, knowledge, confidence, and health behaviours (see Table 2).

**Table 2:** Quantitative outcomes measures to assess app efficacy in the SUCCESS study.

Measure	Description
Health literacy skills	The Health Literacy Questionnaire (HLQ) is a multi-dimensional tool that measures health literacy across nine distinct conceptual domains [29]. We purposefully selected three domains to map to SUCCESS app aims and content: ▪ Domain 2: *‘Have sufficient information to manage my health’* (Model Fit – *χ* ^2^_WLSMV_(2) = 5.24, *p* = 0.0730; Composite reliability = 0.88 (0.87–0.90)) ▪ Domain 6: *‘Ability to actively engage with healthcare providers’* (Model Fit – *χ* ^2^_WLSMV_(5) = 74.91, *p* < 0.0001; Composite reliability = 0.90 (0.88–0.92)) ▪ Domain 9: *‘Understand health information well enough to know what to do’* (Model Fit – *χ* ^2^_WLSMV_(5) = 35.70, p < 0.0001; Composite reliability = 0.88 (0.86–0.90)) Domain 2 assesses the strength of a participant's agreement with the statement (four-point likert scale: 1 = *strongly disagree,* 4 = *strongly agree*), while domains 6 and 9 assess a participant's perceived ease in task completion (five-point likert scale: 1 = *always difficult,* 5 = *very easy*).
Decision self-efficacy	The Decision Self-Efficacy Scale measures self-confidence or belief in one's ability to make informed health decisions, including participating in shared decision-making [30]. Participants rate their confidence engaging in 11 decision-making behaviours, such as ‘*Getting the facts about the medication choices available’* (five-point ordinal scale: 1 = *not at all confident,* 5 = *very confident*). For interpretation purposes, the items were summed, divided by 11 and then multiplied by 25. Scores range from 0 to 11 where the higher the score the greater self-efficacy. The alpha coefficient of the Decision Self-Efficacy Scale is 0.92. The scale has been found to be correlated with: decisional conflict subscales of feeling informed (*r* = 0.47) and supported (*r* = 0.45) [30].
Quality of life	The Kidney Disease Quality of Life (KDQOL-36) is a self-report measure developed for individuals with CKD receiving dialysis treatment [31]. It assesses general health-related quality of life including the Short-Form 12 instrument (SF-12) and three kidney-specific measures: a) *burden of kidney disease*; b) *symptoms*; c) *effects of kidney disease*. Scores range from 0 to 100 where a higher score correlates to greater disease burden. A validation study of the KDQOL-36™ among Hispanic and Non-Hispanics living with Chronic Kidney Disease in the US found that reliability of each KDQOL-36™ subscale was very good (Cronbach's alpha >0.8) [32].
Health behaviour	A theory-informed 11-item behaviour questionnaire adapted from previous literature [33] and matched to the content of the SUCCESS app. Patients respond with *‘yes’* or *‘no’* to engaging in behaviours over the past week, such as: *‘Checking the nutrition label when eating packaged food’* (0 = *no*, 1 = *yes*).
Knowledge	An eight-item curriculum-based measure to assess knowledge about four key topics of dialysis self-management covered in the SUCCESS app: a) *diet*; b) *fluids*; c) *medicines*; d) *physical activity*. Questions (two per topic) were based on key learnings from the app, with points scored for correct answers (0 = *incorrect*, 1 = *correct*). Missing responses were scored 0 and a total score was calculated (range 0 to 8).
Confidence	An 11-item confidence measure based on the health behaviour questionnaire above, including questions such as: *‘How confident do you feel reading and understanding food labels?’* (five-point ordinal scale: 1 = *not at all confident* and 5 = *extremely confident)*. An average was calculated for the 11 items.

A modified version of the mHealth App Usability Questionnaire (MAUQ) [28] was included at the end of the follow-up questionnaires to examine usability of the SUCCESS app. The MAUQ tool was developed to evaluate usability of an mHealth app before it is released to the public. We modified the MAUQ to include 8 questions relevant to our study (see Box 2).

Box 2 **Modified MAUQ used to evaluate usability of the SUCCESS app after 12 weeks.**

**Table:** 

Rate the following on a scale of 1 to 7 where 1 = No and 7 = Yes: 1. Do you think this app is useful for your health and wellbeing? 2. Does the app improve your access to healthcare services? 3. Does the app help you manage your health effectively? 4. Would you feel comfortable using this app in a social setting? 5. Do you think the amount of time involved in using the app suits your lifestyle? 6. Would you use this app again? 7. Would you recommend it to a friend? 8. Overall, are you satisfied with the app?

Alt-text: Box 2

Data was initially collected using paper-forms but for ease of collection and storage we transitioned to REDCap, a digital data platform accessible from smartphones and tablets. This became especially useful during the COVID-19 pandemic, allowing a relatively smooth transition to remote data collection, with research team assistance over the phone as necessary.

A significant barrier to data collection was the time taken to complete questionnaires during dialysis sessions. Participants struggled to remain engaged for the full length of the sequential questionnaires which initially took up to 60 min, sometimes resulting in participant withdrawal. To alleviate physical and cognitive load and decrease attrition rates, we reduced questionnaire length. For example, the Knowledge questionnaire was shortened from 20 to eight questions (two per topic: diet, fluids, medications, physical activity). Researchers also offered to return on alternative days to reduce participant fatigue.

For participants with limited vision and dexterity, researchers assisted with completing questionnaires. While this helped to include participants with a broader range of ability, it extended the length of the data collection process. This was particularly relevant before the transition to digital data collection, which was often easier for this group of patients.

### 2.5 Qualitative interviews

Acceptability of the app and participant satisfaction were explored through semi-structured interviews conducted upon completion of follow-up questionnaires at 12 weeks. Purposive sampling was used to identify patients with broad demographics including age, sex, cultural and linguistic background, and dialysis vintage. Interviews were optional and framed as an opportunity for participants to provide feedback about the app for future improvements.

Interviews were conducted by research staff trained in qualitative methods (SZ, KS, JT, WQ and FV). Interviews took place during dialysis sessions and were completed immediately after follow-up questionnaires, or on separate occasions, depending on participant fatigue. Interviews were typically 20–30 min in duration.

Interview guides were iteratively developed with input from all authors and included open-ended questions regarding app usage, content preferences, usability and suggestions for improvements (see Appendix).

All qualitative data were analysed using the Framework approach to thematic analysis as described by Ritchie and colleagues [34]. This is an inductive approach with an iterative review process, allowing for emergence of core themes through analysis and discussion of data. See Table 3 for more details of this process.

**Table 3:** Qualitative data analysis using the five key steps of the Framework approach.

Key steps	Approach*
Familiarisation	JI, SZ and DM independently read a random selection of transcripts to iteratively develop a preliminary coding scheme over a series of discussions. An inductive orientation was emphasised during this phase, in that most codes referred to semantic or descriptive elements of the data, such as sections of the app that were most frequently accessed and participant suggestions for app improvement [35].
Creating a thematic framework	JI, SZ and DM developed an initial thematic framework from the codes developed in the familiarisation stage. At the point of collating codes, the emphasis shifted to a more reflexive thematic analysis (with a stronger deductive orientation). This allowed the analysts to draw on their expertise in psychology, medicine and public health and shift away from descriptions to focus on patterns of assumptions and meaning [35].
Indexing	JI and SZ coded transcripts according to the framework, with new themes and revisions discussed with DM.
Charting	JI and SZ summarized themes and supporting quotes from each transcript in the framework (a matrix developed in Microsoft Excel with participants as rows and themes as columns).
Mapping and interpretation	JI and SZ examined the framework within and across themes and participants, to identify overarching themes and relationships, and discussed their interpretations with DM.

* Ritchie J, Lewis J. Qualitative research practice: a guide for social science students and researchers. London, England: Sage; 2003.

### 2.6 Quantitative analysis

As this was a feasibility study, a power calculation was not performed to determine sample size [36]. A pragmatic target of 150 was set prior to the study. Descriptive statistics were used to summarise demographic characteristics and study outcomes, with paired sample *t-tests* used to assess change in outcomes pre- and post-intervention. We used Chi square tests to compare demographic characteristics between participants who completed follow-up questionnaires and those who did not. All data were analysed using IBM SPSS Version 25 with *P* < 0.05 considered statistically significant. Missing data were excluded pairwise from the analysis.

## 3 Results

In total, 122 participants were recruited between May 2019 and September 2020. This was 28 participants short of the recruitment target (*n* = 150). However, the study incurred significant delays from March to July 2020 due to the COVID-19 pandemic, during which research activities were paused under mandate.

**Fig. 1:**
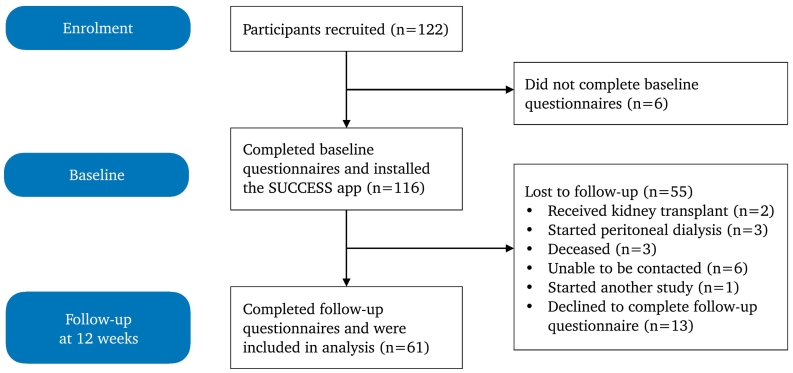
Flow of participants through the study.

Of the recruited participants, 6 were excluded from the analysis as they did not complete baseline questionnaires. An additional 55 did not complete follow-up questionnaires, leaving a sample of 61 (50%) participants included in the analysis (Fig. 1). Unfortunately, the majority of participants did not provide reasons for not completing follow-up questionnaires.

Analysis of field notes and recruitment logs suggested recruitment was easiest when dialysis nurses helped to approach patients due to pre-existing relationships. Engaging older participants with technological hesitancy was challenging but overcome by clear demonstrations of the app and help from carers as needed.

Dialysis units were challenging environments for recruitment due to regular visits by nursing staff and loud noises from dialysis machines. This was compounded by malaise, lethargy and cognitive impairment experienced by many patients. However, the environment was also opportunistic given that dialysis treatments typically occur three times a week for four-to-five hours, during which patients remain seated and are often looking for distractions. These issues highlighted the need for flexibility in recruitment methods, including being comfortable with interruptions, and pausing to return another time if participants had competing needs.

### 3.1 Sample characteristics

The demographic characteristics of participants are summarised in Table 4. Our recruited sample was representative of Australia's in-centre dialysis population, with approximately 60% males and majority aged over 55 [37]. On average, the sample displayed mild cognitive impairment (MoCA score: M = 23.0 ± SD = 4.4, *n* = 116). Participants who did not complete follow-up questionnaires displayed significantly greater cognitive impairment (MoCA score: 22.1 ± 5.0, *n* = 55) than those who did complete them (MoCA score: 23.8 ± 3.8, *n* = 61) (*p* = 0.04). There were no additional statistically significant differences between those who completed the study and those who did not, on any other demographic variable.

**Table 4:** Characteristics of participants recruited to the SUCCESS feasibility study.

Demographic characteristic*	All Sample (*n* = 116)	Completed study (*n* = 61)	Did not complete (*n* = 55)	*P*-value
Age (years) (M ± SD)	59.1 ± 14.6	57.2 ± 14.6	61.2 ± 14.5	0.14
Cognitive impairment (MoCA) (M ± SD)	23.0 ± 4.4	23.8 ± 3.8	22.1 ± 5.0	0.04
Gender (male)	74 (63.8)	42 (68.9)	32 (58.2)	0.23
Country of birth				0.62
*Australia*	64 (55.2)	35 (57.4)	29 (52.7)	
*Other*	52 (44.8)	26 (42.6)	26 (47.3)	
Years since arrival in Australia				0.12
*<5 years*	0 (0.0)	0 (0.0)	0 (0.0)	
*5–9 years*	2 (3.8)	2 (8.0)	0 (0.0)	
*10–14 years*	6 (11.5)	4 (16.0)	2 (7.4)	
*15–19 years*	3 (5.8)	0 (0)	3 (11.1)	
*>20 years*	41 (78.8)	19 (76.0)	22 (81.5)	
Language spoken at home				0.19
*English*	68 (57)	37 (60.7)	31 (56.4)	
*Other*	48 (43)	24 (39.3)	24 (43.6)	
Aboriginal or Torres Strait Islander				0.21
*Yes*	5 (4.3)	4 (6.6)	1 (1.8)	
*No*	111 (95.7)	57 (93.4)	54 (98.2)	
Age completed full time schooling				0.77
*<15 years old*	19 (16.4)	10 (16.4)	9 (16.4)	
*16 years old*	27 (23.3)	13 (21.3)	14 (25.5)	
*17–18 years old*	37 (31.9)	22 (36.1)	15 (27.3)	
*>18 years old*	33 (28.4)	16 (26.2)	17 (30.9)	
Highest level of education				0.32
*None*	37 (31.9)	18 (29.5)	19 (34.5)	
*Certificate*	32 (27.6)	21 (34.4)	11 (20.0)	
*Advanced Diploma and Diploma*	16 (13.8)	5 (8.2)	11 (20.0)	
*Bachelor Degree*	19 (16.4)	9 (14.8)	10 (18.2)	
*Graduate Diploma/Certificate*	3 (2.6)	2 (3.3)	1 (1.8)	
*Postgraduate Degree*	8 (6.9)	5 (8.2)	3 (5.5)	
*Other*	1 (0.9)	1 (1.6)	0 (0.0)	
Years on dialysis				0.65
*<1 year*	45 (39.1)	23 (37.7)	22 (40.7)	
*1–5 years*	39 (33.9)	24 (29.3)	15 (27.8)	
*6–10 years*	17 (14.8)	7 (11.5)	10 (18.5)	
*10–15 years*	7 (6.1)	3 (4.9)	4 (7.4)	
*>15 years*	7 (6.1)	4 (6.6)	3 (5.6)	
Health Literacy – Self report^+^ [23]				0.20
*Low High*	21 (19.3) 88 (80.7)	14 (23.7) 45 (76.3)	7 (14.0) 43 (86.0)	
Health Literacy – Performance-based^^^ [25]				0.06
*Low*	30 (28.3)	11 (19.3)	19 (38.8)	
*Medium*	24 (22.6)	13 (22.8)	11 (22.4)	
*High*	52 (49.1)	33 (57.9)	19 (38.8)	

M, mean; SD, standard deviation, *row n and (%) unless otherwise stated.

^+^Brief Health Literacy Screener, where scores of 3/5 or less are considered ‘low’ [23].

^^^Bostock & Steptoe functional health literacy measure, where scores of 4/4 are considered ‘high’, 3/4 ‘medium’ and 2/4 or less ‘low’ [25].

Of those of who completed the study, 43% were born in a country other than Australia with the majority living in Australia for over 20 years (76%). Twenty-four participants (39%) reported speaking a language other than English (LOTE) at home and four participants (7%) identified as Aboriginal or Torres Strait Islander. Only 16 participants (26%) had university level education or higher. Approximately one quarter (24%) had low health literacy as assessed by a self-report, single-item screener. Using a performance-based instrument, 42% were identified as having ‘low’ or ‘medium’ health literacy.

Participants who completed the study self-reported a high level of digital literacy using the adapted MACL. On average, participants could *‘switch on and off their mobile phone independently’* (4.9 ± 0.9) and reported that they *‘could do about anything they need to with their mobile phone’* (4.0 ± 1.2). On average, most participants could *‘connect to WiFi’* (4.1 ± 1.5), *‘download apps independently’* (4.0 ± 1.5) and *‘delete apps’* from their phones (4.1 ± 1.4).

### 3.2 Qualitative findings

Eighteen participants provided feedback about their use of the SUCCESS app via qualitative interviews. Generally, participants reported using apps for functional tasks such as banking, social media and entertainment with few using health-related apps other than SUCCESS. Overall, simplicity and ease-of-use were features of apps considered to be most important.

Relating to the SUCCESS app, we identified three major themes from our analysis: i) accessed and useful content: the importance of perceived relevance, ii) target audience: relevance across time and dialysis vintage, and iii) ongoing engagement. Perceived relevance was a cross-cutting theme and appeared to be a key mediator of app use.

#### 3.2.1 Accessed and useful content: the importance of perceived relevance

Discussions about useful and frequently accessed content within the SUCCESS app were underpinned by the concept of relevance. Participants reported most frequently accessing the Health Information section, particularly information on diet and fluids. They suggested that these sections reflected the most challenging aspects of living with kidney failure and were perceived to be relevant to the daily management of their health: *Mainly the… fluid one. ‘Cause… I find it hard to like keep to my fluid restrictions. I mean it's easy to drink too much (laughs)* (20–25 years, male, dialysis vintage ≤1 year). This was also the case within content sub-sections of the app. For example, within the Diet section, participants reported accessing information on potassium, phosphates and salt most frequently as they felt it was most relevant to their lived experience.

In contrast, few participants reported using the Skills for Health section, most often because they perceived that they ‘already knew’ how to look after their health. Participants suggested that this content was less personally relevant, novel or useful to daily living compared to content-based information: *For me, I already knew that kind of stuff, so I had a look at it and it was like, yeah, there's nothing really new here for me* (45–50 years, female, dialysis vintage 6–10 years).

Interestingly, however, there was some dissonance between participants' perceived relevance of sections of the app and the skills they felt they needed. For example, one participant said they do not need to use the app because *“if I have a problem with my medicine I will just speak to the doctor”* (50–55 years, female, dialysis vintage >15 years, language other than English (LOTE) at home). However, later in the conversation they noted that doctors often do not provide sufficient information regarding medication side effects, so “*you have to go and look it up sometimes to get the real in depth*”, indicating an area of self-management that could benefit from additional health literacy support.

A minority of participants reported little to no use of the app. For such patients, this was often attributed to having lived with kidney disease for a long time: *I'm not going to change my habits now* (55–60 years, male, dialysis vintage >15 years). Other barriers to app use included limited time, forgetfulness and competing priorities, reflecting the difficulty of engaging this patient population in research: *I have a lot of appointments at the moment… I don't have time to look at the apps* (50–55 years, female, dialysis vintage ≤1 year).

#### 3.2.2 Target audience: relevance across time and dialysis vintage

Perceptions about the app's target audience varied and was influenced by whether the app was considered relevant to the individual. Several participants commented that the SUCCESS app was most useful when first downloaded: *It's a great introduction app, but when it comes to maintaining your diet and such, … after a few months there's no real need for it* (30–35 years, female, dialysis vintage ≤1 year, LOTE at home). For others, however, there was benefit in having an accessible resource to return to multiple times. This was particularly relevant given the struggles with memory and cognition that many dialysis patients suffer: *I always go back, cause my memory's not all there* (30–35 years, male, dialysis vintage 1–5 years, LOTE at home).

Some participants found the information redundant due to the presence of allied health practitioners in the dialysis unit. For example, one participant indicated there was a dietitian who “*comes to talk to us all the time… that's where we ask questions*” (30–35 years, female, dialysis vintage ≤1 year, LOTE at home). In contrast, another participant found the combination of information from the app and the dietitian helpful to reinforce skills and health information: *This sort of just, um, reinforced it you know? Oh I'm being told by the app and I'm being told by her too* (40–45 years, female, dialysis vintage 1–5 years).

One participant who was new to dialysis outlined the utility of having the app to refer back to and clarify information provided by doctors, during what is often an overwhelming initiation into treatment: *I was fairly new to having dialysis when you installed that, so there were questions that sometimes the doctors had gone over with me but, you know, my brain didn't exactly remember what they said so then I'd go back into the app so that I could sort of get clarity with that* (60–65 years, male, dialysis vintage ≤1 year). This sentiment was echoed by multiple participants who suggested that the app would be particularly beneficial for new dialysis patients: *I've been doing this for a very long time… but when you're like new and you don't know who to go to… it's really important to have some sort of um, guidelines* (50–55 years, female, dialysis vintage >15 years, LOTE at home).

#### 3.2.3 Ongoing engagement

There was consensus that for participants to continue engaging with the app, a number of functional and content-related changes needed to be made. Functional suggestions included making a *“fully integrated system*” (50–55 years, female, dialysis vintage ≤1 years, LOTE at home) connected to other apps such as Google Calendar “*cause that means you've got all your information in one, you're not going to different apps to get it”* (60–65 years, female, dialysis vintage ≤1 year). Content suggestions included adding a mental health section incorporating peer stories to learn about *“things that have worked in the past with other patients”* (30–35 years, female, dialysis vintage ≤1 year). A full list of recommendations is provided in Table 5.

**Table 5:** Recommendations to improve the SUCCESS app obtained through qualitative interviews.

Content information	Functional suggestions
• Food and drinks to avoid • How CKD affects the heart • Renal recipes / cookbook • What to expect when starting dialysis • Managing fatigue • Transplant • When to go to ED / emergency situations • Uric acid and gout control • Exercise • Mental health with peer stories	• Instructions for how to use the app • SUCCESS icon more informative • Search bar • More pictures • Notifications to encourage app use • Alarm reminders for plans/calendar • Features (quiz, plans, calendar) more prominent and easy to use • Reduce number of quiz questions • Customisable profile to track individual data over time (eg. meds, BMI, fluids) • Ability to leave a question and have an answer emailed to you by a professional • Translate to other languages • Frequently asked questions

CKD, chronic kidney disease; ED, emergency department; BMI, body mass index.

### 3.3 Quantitative findings

Participants' mean baseline, follow-up and mean score changes for the HLQ domains, Decision Self-Efficacy Scale and KDQOL-36 are presented in Table 6. At follow-up, we did not observe any significant changes in the HLQ domains *‘Having sufficient information to manage my health’* and *‘Understanding health information well enough to know what to do’*. However, we did observe significant changes in the *‘Ability to actively engage with healthcare providers’* at follow-up (*p* = 0.03). In addition, Decision Self-Efficacy scores significantly improved at follow-up (*p* = 0.02*)*. The symptoms, effects of kidney disease, burden of kidney disease and SF-12 Mental Composite of the KDQOL-36 did not change from baseline, however, SF-12 Physical Composite significantly increased (*p* < 0.01).

**Table 6:** Change in health literacy, decision self-efficacy and quality of life from baseline to follow-up (*n* = 60).

Measure	Baseline	Follow-up	Change (follow-up – baseline)	95% CI	*P*-value
Health Literacy Questionnaire Domains	M (SD)	M (SD)	M (SD)		
*Having sufficient information to manage my health (range: 1–4)*^++^	3.1 (0.6)	3.1 (0.6)	0.0 (0.8)	−0.1, 0.2	0.77
*Understanding health information well enough to know what to do (range: 1–5)*^++^ *Ability to actively engage with healthcare providers (range: 1–5)*^++^	3.9 (0.9) 3.9 (0.8)	4.1 (0.8) 4.2 (0.7)	0.2 (1.0) 0.2 (0.8)	−0.0, 0.5 0.0, 0.4	0.11 0.03
Decision Self Efficacy Scale					
*Score (0 not at all confident; 100 very confident)*	80.8 (18.4)	85.1 (15.0)	4.3 (14.1)	0.6, 7.9	0.02
Quality of Life					
*Symptoms (range: 0–100)*^⁎^	72.0^+^ (16.7)	71.9^⁎^ (14.1)	−0.3 (14.9)	−4.5, 4.0	0.90
*Effects of kidney disease (range: 0–100)*^^^	62.4^+^ (23.7)	66.7^^^ (20.7)	4.2 (22.5)	−1.7, 10.1	0.16
*Burden of kidney disease (range: 0–100)*^+^	36.7 (23.3)	38.9^+^ (25.6)	2.8 (22.4)	−2.9, 8.6	0.34
*SF-12 Physical Composite (range: 0–100)*^^^	33.0 (10.6)	36.9^^^ (10.7)	4.4 (8.5)	2.1, 6.6	0.00
*SF-12 Mental Composite (range: 0–100)*^^^	46.7 (11.1)	46.7^^^ (8.8)	0.1 (10.0)	−2.5, 2.8	0.91

M, mean; SD, standard deviation; CI, confidence interval; ^++^missing 2, ^^^missing 3, ^⁎^missing 13.

At baseline, less than half the participants engaged in health behaviours such as *‘Using strategies to change my diet’* (45%), *‘Completing 30 minutes of exercise most days’* (43%), *‘Using strategies to do more exercise’* (38%) and ‘*Checking whether I could trust online information*’ (48%) (Table 7). While not statistically significant, at follow-up participants reported increased engagement in the following behaviours: *‘Checking nutrition labels’* (11% increase), *‘Completing 30* min *of exercise most days’* (8% increase) and ‘*Using strategies to do more exercise’* (5% increase).

**Table 7:** Change in health behaviours, knowledge and confidence from baseline to follow-up (*n* = 61).

**Health Behaviours**	**Baseline N (%)**	**Follow-up N (%)**	**Change (%)**
Check nutrition labels	31 (50)	37 (60.7)	10.7
Use strategies to change diet	28 (45.2)	28 (45.9)	0.7
Do 30 min of exercise most days	26 (42.6)	31 (50.8)	8.2
Use strategies to do more exercise	23 (37.7)	26 (42.6)	4.9
Keep track of fluids	44 (72.1)	44 (72.1)	0.0
Use strategies to reduce fluids	38 (62.3)	38 (62.3)	0.0
Check medicine labels	37 (60.7)	37 (60.7)	0.0
Use strategies to remember to take medicine	32 (52.5)	27 (44.3)	−8.2
Ask HCT questions	38 (62.3)	33 (54.1)	−8.2
Talk to HCT about what matters to you	37 (60.7)	34 (55.7)	−5
Check trustworthiness of online information	29 (47.5)	26 (42.6)	−4.9

**Knowledge**	**Baseline N (%) correct**	**Follow-up N (%) correct**	**Change (%)**

Diet			
Which product has less salt per serve?	46^+^ (76.7)	42 (68.9)	−8.1
Which packet should you choose for lower salt?	36^+^ (60.0)	34 (55.7)	−5
Physical Activity			
How many minutes of moderate intensity physical activity is recommended per day?	38^!^ (76)	44^^^ (75.9)	−0.1
Which of the following is not considered moderate intensity physical activity?	20 (40)	25^^^ (43.1)	3.1
Medicine			
How many tables should you have each day?	55^+^ (91.7)	51^+^ (83.6)	−8.2
When would be the best time for Anna to take her first tablet each day?	48^+^ (80.0)	45^+^ (73.8)	−4.9
Fluids			
True or false: Drinking too much fluid causes swelling puffiness in the body (face, legs, feet).	58 (95.1)	60 (98.4)	3.2
True or false: Drinking more than 3 l between your dialysis sessions is harmful.	57 (93.4)	58 (95.1)	1.6
Knowledge Total Score (*out of 8;* M(SD))	5.9^+^ (1.5)	5.9^+^ (1.6)	0.2 (1.8)

**Confidence** (range 1–5)	**M (SD)**	**M (SD)**	**M (SD)**

Check nutrition labels	3.9 (1.2)	4.2 (0.9)	0.3 (1.1)
Choose healthy meals	3.7 (1.1)	4.1 (0.8)	0.3 (1.1)
Keep track of fluids	4.0 (1.2)	3.9 (1.2)	−0.0 (1.0)
Use strategies to reduce fluids	3.7 (1.2)	3.5 (1.3)	−0.1 (1.3)
Make a plan to do more exercise	3.1 (1.3)	3.1 (1.4)	0.0 (1.8)
Know when to stop exercising	3.6 (1.2)	3.5 (1.4)	0.0 (1.7)
Check medicine labels	3.8 (1.4)	4.1 (1.1)	0.3 (0.9)
Take the correct dosage of medicines	4.6 (0.6)	4.6 (0.7)	−0.0 (0.7)
Share decisions with HCT	4.0 (1.0)	4.2 (0.8)	0.2 (1.1)
Talk to HCT about what matters to you	3.9 (1.0)	4.3 (0.9)	0.4 (1.2)
Check trustworthiness of online information	3.1 (1.3)	3.2 (1.4)	0.1 (1.4)
Confidence Average *(11 items)*	3.8^+^ (0.6)	3.9^+^ (0.6)	0.1 (0.6)

HCT, healthcare team; M, mean; SD, standard deviation; ^+^missing 1, ^++^ missing 2, ^^^missing 3,^!^missing 11.

Overall, we did not observe significant improvements in the average total score for knowledge (baseline: M = 5.9 ± SD = 1.5 out of 8, follow-up: 5.9 ± 1.6, *p* = 0.943) or confidence measures (baseline: 3.8 ± 0.6 out of 5, follow-up: 3.9 ± 0.6, *p* = 0.24) (Table 7). At both baseline and follow-up, average knowledge scores were highest in relation to fluids and medicine, and lowest for diet and physical activity. At both baseline and follow-up, average confidence scores were highest for tasks related to keeping track of daily fluids (4.0 ± 1.2), medicine dosage (4.6 ± 0.6) and sharing decisions with their healthcare team (4.0 ± 1.0). Average confidence scores were lowest for making exercise plans (3.1 ± 1.3) and critically appraising online information (3.1 ± 1.3).

42 participants completed the MAUQ and most participants agreed that the app was *‘useful for their health and wellbeing’* (mean [M] = 6 on a seven-point scale, inter-quartile range [IQR]: 3), *‘felt comfortable using it’* (M = 6; IQR: 2), *‘would use it again’* (M = 6; IQR: 3), *‘felt the amount of time involved in the app suited their lifestyle’* (M = 6; IQR: 2), overall were *‘satisfied with the app’* (M = 6; IQR: 3) and strongly agreed that they would *‘recommend it to a friend’* (M = 7; IQR: 2). Participants neither agreed or disagreed that the app could *‘improve their access to healthcare services’* (M = 4; IQR: 3), and somewhat agreed that the app could *‘help manage their health’* (M = 5; IQR: 3).

## 4 Discussion and conclusion

### 4.1 Discussion

The delivery of the SUCCESS app across four Local Health Districts in New South Wales, Australia suggests it was feasible to recruit a broad sample of haemodialysis patients from diverse cultural backgrounds with varying levels of health literacy. Interest to participate in the study was high with 116 participants completing baseline questionnaires. Ongoing engagement and retention were more challenging; only 61 participants completed all follow-up questionnaires, with statistically greater cognitive impairment in those who were lost to follow-up. The extent to which the COVID-19 pandemic affected the attrition rate is unclear. Statistically significant increases in decision self-efficacy and certain domains of health literacy and quality of life offer encouraging insights into app efficacy, warranting further attention in an adequately-powered trial.

As with several other feasibility studies conducted in the chronic disease context [38,39], the main feasibility outcomes in this study included recruitment and retention of participants. We were also interested in exploring the feasibility of collecting data during scheduled dialysis sessions. A strength of this study was the successful engagement of a culturally and linguistically diverse range of participants. With help from dialysis nurses, we recruited participants from 29 countries, 55% of whom spoke a language other than English at home. Only one quarter of our study sample attained university level education, and at least one quarter was identified as having low health literacy. This is in contrast with many other chronic disease research studies, which often fail to recruit diverse cohorts [40].

In regards to data collection, participant fatigue required researchers to return multiple times to complete questionnaires, with exhaustion sometimes resulting in participant withdrawal from the study. Fatigue and reduced concentration are common for haemodialysis patients [41]. A cross-platform app modality was specifically chosen to help reduce the cognitive burden for participants by providing small unit-based learning allowing users to return to the content in their own time [17]. This appeared to be useful for many participants, as reported by our qualitative data. In addition, recruitment logs suggested that reducing questionnaire lengths led to a decrease in participant withdrawal. Although our attrition rate was high (47%) it is similar to the reported pooled attrition rate from other e-health interventions for chronic conditions [42].

This feasibility study highlighted important challenges regarding the SUCCESS app to address in future trials. Qualitative analyses suggested that perceived relevance was a key mediator of app use, whereby participants displayed strong preferences for health information (e.g. diet and fluids) over skills relating to health literacy and shared decision-making. As such, a key goal for future iterations of the SUCCESS app will be to enhance the delivery of the ‘Skills for Health’ content. This may be through redesigning the app to increase usability via targeted notifications/reminders, signposting to increase understanding of content relevance and/or greater integration of health literacy skills training within content which is perceived as relevant and desired by patients. Previous research has similarly identified the need to embed health literacy skills training into health topics of interest. Iterative revisions of the Parenting Plus health literacy intervention for new parents, for example, were required to achieve an acceptable balance between health literacy skills and content-specific health knowledge [43]. Additionally, this feasibility study also reinforced the need for content that caters to all levels of dialysis experience, with potential to customise information based on individual needs. Delivery of interactive features such as quizzes, plans, calendar and questions, which were minimally accessed, will be re-evaluated using participants' suggestions to enhance content delivery and promote ongoing engagement over time.

As a feasibility study with a relatively short follow-up period, this study was not designed to detect significant pre- or post-test differences in health outcomes. Rather, it aimed to provide meaningful insights into how the app and study procedures can be adapted and optimised. A mixed methods approach including both qualitative and quantitative data collection helped to achieve these aims. To ensure rigor in qualitative analysis, all data were indexed by at least two researchers, with continuous comparison of concepts and themes. Comprehensive data collection and field logs enabled real-time adjustments to recruitment processes, informed by participant feedback and experiences. While the SUCCESS intervention is targeted toward culturally-diverse patients, a basic requirement for enrolment in this feasibility study was basic English proficiency. Future plans for the SUCCESS app include cultural adaptation and translation processes.

### 4.2 Innovation

There is growing evidence linking the usage of mobile apps and web-based tools to improved clinical outcomes [[9], [10], [11], [12], [13], [14], [15]]. However, few available apps support self-management specifically for people with CKD [16], and none have been informed by health literacy theory. Furthermore, many CKD-related apps lack accurate and evidence-based information [44]. The SUCCESS app adds innovation in the e-health space by tailoring content about self-management and shared decision-making to culturally-diverse and low health literacy haemodialysis patients. This is important, given that previous research from our group showed that individuals from these population groups are often unengaged in decision-making about their health [45].

## 5 Conclusion

Enhancing health literacy skills for haemodialysis patients is an important endeavour given the association between poor health literacy and poor health outcomes, especially among culturally-diverse groups. Findings from this study suggest it was feasible to recruit a diverse population, and that the SUCCESS app intervention was acceptable and may have the potential to engage patients in self-management and shared decision-making. This is particularly important in the era of telehealth, with increasing emphasis on empowering patients to self-manage their chronic health conditions. Perceived relevance was a central mediator of app use and highlighted that the app in its current form may be most relevant for patients commencing dialysis. A key goal will be finding ways to engage those with longer dialysis vintage who may benefit from the content and skills training within the app. Having established feasibility, we are now better placed to incorporate lessons learned and move toward a larger and more structured trial of efficacy.

## Funding

This work was supported by Sydney Health Partners Medical Research Future Fund (MRFF) Rapid Applied Research Translation grant (2019) and 10.13039/501100000975Kidney Health Australia Medical and Scientific Kidney Research Grant [KHA2018-AW].

## Declaration of Competing Interest

None.

## Appendix SUCCESS Qualitative Interview Guide

**Introduction**

*Before we get started, there are a few things I’d like to explain about this conversation.*

1. Discuss confidentiality
2. Explain audio recording
3. There are no right or wrong answers

**App usage**

*Before speaking about the SUCCESS app I’d like to get a sense of how you use apps in general.*

1. Do you use apps on your phone?
  a. *If yes:*
    • Can you tell me about the kinds of apps that you use?
    • What do you like about these apps?
    • What do you use them for?
  b. *If no:*

• What puts you off about using apps?
• What would make you want to use an app more?

*Now I’d like to ask you some specific questions about the SUCCESS app.*

2. Did you use the SUCCESS app?
  a. *If yes:*

• What influenced you to use it?
• How much did you use it?
• In what setting did you use it (eg. during dialysis, at home)?
• Which parts of the app did you use?
• Why did you use those sections?
• What did you like about those sections?
• What did you dislike about those sections?
• Did the app help change your behaviour in any way?
• What was new for you?
  b. *If no:*
• Can you tell me about why you didn’t use the app?
• What didn’t you like about it?
• Was it different from what you expected it would be?
• Was it difficult to use?

3. Is there anything that would make you want to use the app more?
4. Do you think it would be useful for your family/carer to use an app like this?

**Content acceptability and functionality**

*I’m now going to ask you a few questions about the topics in the app.*

1. What do you think about this selection of topics *[show home screen]*?
2. What stands out to you as the most and least important topics?
3. The app has information about: *Diet, Fluids, Medicines, Physical Activity and Treatment Goals*

a. Can you tell me what you think about these topics?
b. Are there any topics you’d like to see that aren’t there?

4. The app also provides skills training. This aims to help you speak to your doctors about your needs and make decisions that suit you best. These sections include: *Thinking about what matters to me; Shared decision making; Can I trust this health information?*
  a. Can you tell me what you think about these topics?
  b. Are there any topics you’d like to see that aren’t there?
5. Are there pieces of information or skills that you’ve picked up during your time on dialysis that you think should be included in the app?
6. The app also includes interactive features such as: *Calendar, Videos, Quizzes, Plans, Links to other websites and apps*
  a. Are these features easy to find?
  b. Did you know they were there?
  c. What do you think of them?
  d. Are they useful?
  e. What other features would you like to see there?
7. What changes could we make to the app to better suit you?

**Conclusion**

*Is there anything else you would like to tell us or ask about the app?*

*Thank you for your time.*
